# Environmental determinants of aplastic anemia in Pakistan: a case-control study

**DOI:** 10.1007/s10389-016-0743-6

**Published:** 2016-06-11

**Authors:** Mehwesh Taj, Tayyaba Shah, Syeda Kanwal Aslam, Sidra Zaheer, Faryal Nawab, Sumaira Shaheen, Kashif Shafique, Tahir Sultan Shamsi

**Affiliations:** 1 Department of Clinical Hematology, National Institute of Blood Disease Center and Bone Marrow Transplantation, Karachi, Pakistan; 2 School of Public Health, Dow University of Health Sciences, Karachi, Pakistan; 3 Department of Research and Development, National Institute of Blood Disease Center and Bone Marrow Transplantation, Karachi, Pakistan; 4 Institute of Health and Wellbeing, Public Health, University of Glasgow, 1-Lilybank Gardens, Glasgow, UK G12 8RZ

**Keywords:** Aplastic anemia, Environmental exposures, Pesticides, Arsenic, Case-control study

## Abstract

**Aim:**

Aplastic anemia (AA) affects the Asian population two to three fold more than people in other regions. Besides the host genetics and socioeconomic status, several other environmental exposures have been linked with an AA etiology. We aimed to examine the association of various environmental exposures with AA occurrence among Pakistani individuals.

**Subjects and methods:**

A case-control study was conducted in Karachi, Pakistan, where cases (diagnosed AA patients) were selected from the National Institute of Blood Disease and Bone Marrow Transplantation (NIBD), while for each case, a single control (who was free of AA and visited the outpatient department of the same hospital for the treatment of minor ailments) was selected matched by age and sex. A total of 428 participants were included in this study with equal proportions of cases and controls. Information related to disease characteristics, sociodemographics and exposure to chemicals was collected through a survey questionnaire, laboratory investigations and medical records. Descriptive results were reported as frequencies and proportions, adjusted odds ratios with 95 % confidence intervals and population attributable risk (PAR) as percentage.

**Results:**

Among study participants (*n* = 428), AA was significantly associated with various environmental exposures. Participants residing in rural settings (OR = 2.29, 95 % CI 1.12–4.67, *p*-value < 0.01) and those who reported exposure to pesticides (OR = 3.58, 95 % CI 1.27–10.10, *p*-value 0.01; PAR = 18.16 %) were significantly more likely to report AA. Participants with a formal education were significantly less likely to have AA (OR = 0.27, 95 % CI 0.10–0.71, *p*-value < 0.01).

**Conclusions:**

This study observed a significant association of aplastic anemia with a lower socioeconomic profile, and certain environmental exposures among the Pakistani population. The evidence may be helpful in understanding the pathophysiology of aplastic anemia in the context of environmental exposures.

## Background

Aplastic anemia (AA) is defined as “bone marrow hypoplasia or aplasia resulting in pancytopenia”; it affects 2–7 million individuals globally (Shadduck 1995; Young and Kaufman 2008). Wide variation in the prevalence of AA has been observed (Young and Kaufman 2008). Individuals in Asian countries are affected two to three fold more than populations from other regions (Kojima 2002; Mary et al. 1990; Young and Kaufman 2008). Scientific evidence suggests that the wide variation in the prevalence of AA among different regions of the world could be due to variations in environmental exposures (Issaragrisil et al. 1997; Maluf et al. 2009; Young and Alter 1994). Asian residents in other regions, for instance, have experienced AA at a rate native to the region of immigration, which favors the environmental predisposition more than the genetic one (Young and Kaufman 2008). As evidence related to AA’s etiology continues to build, there is still a dearth of sound epidemiological evidence regarding the etiological link of environmental exposure to various chemicals with AA. Very few risk factors have thus been identified, and risk of disease development is mostly linked with the host genetics (Young and Kaufman 2008). Among the various chemicals suspected of an etiological association with AA, pesticides, arsenic and benzene have been found to be strongly linked with an increased susceptibility of individuals to develop AA (Beelte et al. 2009; Chatterjee et al. 2014; Fleming and Timmeny 1993; Morton and Dunnette 1994; Peremarti et al. 2014; Prihartono et al. 2011).

Unfortunately, evidence from the developing world, where AA prevalence is higher, is limited, but is called for (Maluf et al. 2009). Further, to the best of our knowledge, no such study reports evidence related to environmental exposures’ etiological link with AA among the Pakistani population. The majority of the studies conducted in Pakistan report evidence related to the clinical and pathological features, genetic susceptibility and association with infectious agents such as hepatitis (Adil et al. 2001; Niazi and Raziq 2011; Rauff et al. 2011; Shamsi et al. 2008; Zahra et al. 2015). We thus aim to explore the association of environmental exposure to various chemicals with AA disease occurrence in the Pakistani population.

## Methods

### Study settings and participants

A case-control study was conducted in the largest metropolitan city, Karachi, in Pakistan. The participants included patients accessing NIBD for healthcare from January 2014 to December 2014.

### Cases

Cases included all patients aged 12 and above with a confirmed diagnosis of aplastic anemia as per the Camitta Criteria (Camitta et al. 1975). Eligible cases included patients who met at least two of the following three criteria: (1) white cells <3.5 × 10^9^/l; (2) platelets <50 × 10^9^/l; (3) hemoglobin <10.0 g/dl or hematocrit <30 %. Further following the criteria set by E. Maluf et al., hypocellularity was shown on bone marrow biopsy, without leukemic, lymphomatous or carcinomatous infiltration or fibrosis (Maluf et al. 2009). All patients were residing in Pakistan at the time of the study. Exclusion criteria included presence of hypocellular myelodysplasia, other severe hematologic diseases such as other neoplasias, neural tube defects, megaloblastic anemia, neutropenia or pancytopenia associated systemic diseases, previous organ transplantation, chemotherapy, radiotherapy or immunosuppressive therapy, Felty’s syndrome or Kostmann’s syndrome. Profiles of all cases were finally validated by consultant hematologists who had access to patients’ files containing the complete clinical history, blood reports and biopsy results.

### Controls

The controls were selected by matching for age and sex. For each case of aplastic anemia, a single control was selected. They consisted of walk-in patients who attended the outpatient department during the study period for ailments other than blood disorders, including minor ailments such as sore throat and diarrhea.

### Data collection tool

Participants’ information was collected through a pretested, structured questionnaire. The same questionnaire was used for both cases and controls. The questionnaire consisted of modules related to (1) sociodemographic characteristics including sex, age, ethnicity, urban/rural type of residence, marital status and education level; (2) eating habits and drinking water source; (3) environmental exposures related to pesticides and arsenic (a pictorial album was used by the interviewer to facilitate participants’ recall of exposures related to pesticides and arsenic). Further, information related to the hematological profile of the patients was retrieved from their medical records. Data were collected by trained clinical research officers.

### Statistical analysis

Data were analyzed using STATA SE version 11.1. Chi-square test was used to determine the significance of association of categorical variables between cases and controls. Univariate and multivariate conditional logistic regression analyses were used, and odds ratios with 95 % confidence intervals were reported to determine an association between exposures and disease occurrence (AA). First, an association between the different forms of environmental exposures and AA was reported in model 0. Subsequently, model 1 reported the results after adjusting for sociodemographic characteristics including type of residence, education level, ethnicity and marital status. Third, model 2 reports the results after adjusting for the type of residence, education level, ethnicity, marital status, water source and source of milk intake. Finally, model 3 reports the results after adjusting for all covariates with the environmental exposures to arsenic and pesticides.

Further, the population attributable risk (PAR) percentage was also calculated for the exposures that were found significantly associated with AA disease occurrence. This may be defined as “the proportional reduction in population disease or mortality that would occur if exposure to a risk factor were reduced to an alternative ideal exposure scenario” [Bruzzi et al. 1985; Health statistics and information systems. Metrics: population-attributable fraction (PAF)].

A subgroup analysis was also performed by matching for additional factors such as marital status, education level and type of residence. Both univariate and multivariate analyses were repeated on this subgroup of the sample to validate the findings obtained from age- and sex-matched analysis.

## Results

A total of 428 individuals were included in the study. Of these, 214 patients had confirmed diagnoses of AA, and 214 were taken as matched controls. Age and sex distributions of cases and controls were similar because of matching (chi-square test *p*-value = 1.00). Among cases of AA, 11.3 % (*n* = 24) were exposed to arsenic, and 25.2 % (*n* = 54) were exposed to pesticides, as compared to only 4.7 % (*n* = 10), 3.7 % (*n* = 8) and 23.3 % (*n* = 50) among controls, respectively. Regarding education status and type of residence, 30.2 % of AA patients (*n* = 65) had no education, and 39.5 % (*n* = 85) of them resided in rural environments, as compared to only 8.9 % (*n* = 19) and 15.3 % (*n* = 33) among controls, respectively. Among AA cases, 84.2 % (*n* = 181) used fresh milk, and only 6 % (*n* = 13) used tetra-pack milk, whereas use of fresh milk was lower among controls, i.e., 74.4 % (*n* = 160) and 13.5 % (*n* = 29) of them used tetra-pack milk. The source of household water supply among 68.4 % (*n* = 147) of the cases was tap water, as compared to 78.6 % (*n* = 169) among controls (Table 1).

**Table 1:** Distribution of cases of aplastic anemia (AA) and controls according to exposure to pesticides and arsenic with sociodemographics (*n* = 428)

Characteristics	Cases	Controls	*p*-value*
	*n* (%)	*n* (%)	
Exposure to pesticides (organophosphates/DDT/insecticides/mosquito repellent)			
No	160 (74.8)	206 (96.3)	<0.01
Yes	54 (25.2)	8 (3.7)	
Exposure to arsenic			
No	190 (88.7)	204 (95.3)	0.01
Yes	24 (11.3)	10 (4.7)	
Source of milk intake			
Fresh milk	179 (84.2)	160 (74.4)	0.04
Tetra-pack	14 (6.0)	28 (13.5)	
Powdered milk	17 (7.9)	21 (9.8)	
Mixed source	4 (1.9)	5 (2.3)	
Water source			
Tap water	146 (68.4)	168 (78.6)	<0.01
Hand pump	44 (20.5)	13 (6.1)	
Mineral/filter	16 (7.4)	31 (14.4)	
River	8 (3.7)	2 (0.9)	
Type of residence			
Urban	130 (60.5)	181 (84.7)	<0.01
Rural	84 (39.5)	33 (15.3)	
Education level			
No education	64 (30.2)	20 (8.9)	<0.01
Primary	59 (27.4)	70 (33.2)	
Secondary	63 (29.3)	73 (34.1)	
Higher	28 (13.1)	51 (23.8)	
Marital status			
Unmarried	152 (70.9)	159 (74.4)	0.44
Married	62 (29.1)	55 (25.6)	
Ethnicity			
Urdu speaking	67 (30.8)	81 (38.1)	<0.01
Sindhi speaking	44 (20.6)	54 (25.1)	
Punjabi speaking	38 (17.8)	38 (17.7)	
Pashtun speaking	44 (21.0)	13 (6.1)	
Balochi speaking	16 (7.5)	19 (8.8)	
Others	5 (2.3)	9 (4.2)	

*p-value calculated using the chi-square test

### Exposure to pesticides

Univariate logistic regression (model 0) showed that exposure to pesticides was significantly associated with AA cases (OR = 7.57, 95 % CI 3.44–16.65, *p*-value < 0.01). After adjusting for important sociodemographic variables such as type of residence, education level, ethnicity and marital status in model 1, exposure to pesticides remained significantly associated with AA cases (OR = 3.73, 95 % CI 1.47–9.42, *p*-value < 0.01). In model 2, when we adjusted for the type of milk consumed and source of drinking water, pesticide exposure remained significantly associated with AA (OR = 3.12, 95 % CI 1.14–8.53, *p*-value 0.02). At the model 3 level, we adjusted for all covariates in the study and found that pesticide exposure remained a significant risk factor for developing AA (OR = 3.42, 95 % CI 1.24–9.47, *p*-value 0.01) (Table 2). The PAR was calculated as 17.83 %.

**Table 2:** Risk estimates of aplastic anemia (AA) with exposure to environmental factors (*n* = 428)

Characteristics	Model 0		Model 1		Model 2		Model 3	
	OR (95 % CI)	*p*-value	OR (95 % CI)	*p*-value	OR (95 % CI)	*p*-value	OR (95 % CI)	*p*-value
Exposure to pesticides (organophosphates/DDT/insecticides/mosquito repellent)								
No	1		1		1		1	
Yes	7.57 (3.44–16.65)	<0.01	3.73 (1.47–9.42)	<0.01	3.12 (1.14–8.53)	0.02	3.42 (1.24–9.47)	0.01
Exposure to arsenic								
No	1		1		1		1	
Yes	2.74 (1.22–6.17)	0.01	2.05 (0.69–6.09)	0.19	1.75 (0.55–5.57)	0.34	2.18 (0.64–7.43)	0.21

Model 0 = univariate analysis

Model 1 = model 0 + sociodemographics (type of residence, education level, marital status, ethnicity)

Model 2 = model 1 + source of milk intake + water source

Model 3 = model 0 + model 1 + model 2

### Exposure to arsenic

Univariate logistic regression (model 0) showed that exposure to arsenic was significantly associated with AA cases (OR = 2.74, 95 % CI 1.22–6.17, *p*-value 0.01). After adjusting for important sociodemographic variables such as the type of residence, education level, ethnicity and marital status in model 1, exposure to arsenic remained positively associated with AA cases (OR = 2.05, 95 % CI 0.69–6.09, *p*-value 0.19). In model 2, we adjusted for the type of milk consumed and source of drinking water, and arsenic exposure remained positively associated with AA cases (OR = 1.75, 95 % CI 0.55–5.57, *p*-value 0.34). At the model 3 level, we adjusted for all covariates in the study and found that the odds of having AA increased (OR = 2.18, 95 % CI 0.64–7.43, *p*-value 0.21) (Table 2).

**Fig. 1:**
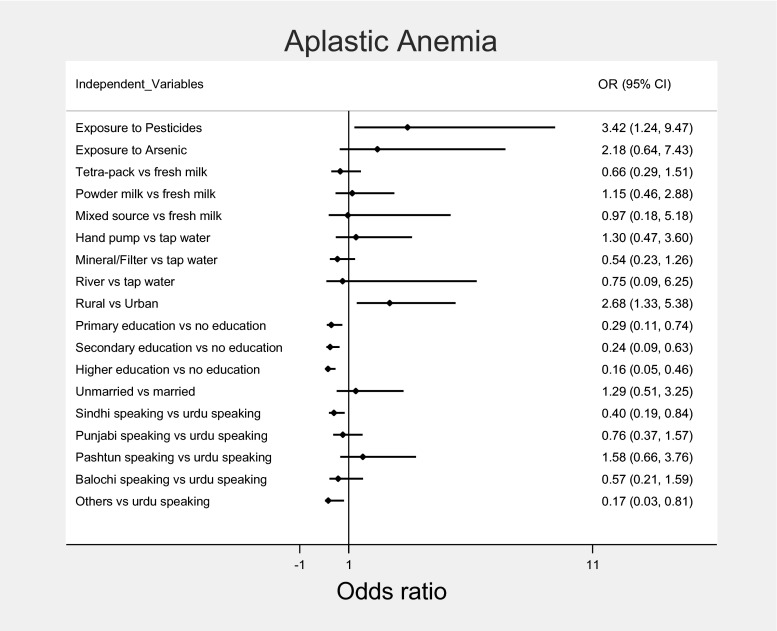
Forest plot showing the random effect of each variable against aplastic anemia (AA) after adjusting for all covariates

After adjusting for all covariates in model 3, we plotted the random effect of each variable against AA through forest plots. We found that rural residents were significantly associated with AA cases as compared to urban residents (OR = 2.68, 95 % CI 1.33–5.38, *p*-value 0.01). Regarding education status, having primary education (OR = 0.29, 95 % CI 0.11–0.74, *p*-value < 0.01), secondary education (OR = 0.24, 95 % CI 0.09–0.63, *p*-value < 0.01) and higher education (OR = 0.16, 95 % CI 0.05–0.46, *p*-value < 0.01) was found to be significantly protective against AA (see Fig. 1).

Furthermore, a subgroup analysis was also performed after matching for age, gender, type of residence, education level and marital status. The basic demographics of this subgroup analysis with additional matching are shown in Table 3. A total of 194 individuals were included in the subanalysis, 97 patients had confirmed diagnoses of AA, and 97 were taken as matched controls for age, sex, education level, type of residence and marital status.

**Table 3:** Distribution of cases of aplastic anemia (AA) and controls according to exposure to pesticides and arsenic with sociodemgraphics (*n* = 194)

Characteristics	Cases	Controls	*p*-value*
	*n* (%)	*n* (%)	
Exposure to pesticides (organophosphates/DDT/insecticides/mosquito repellent)			
No	80 (82.5)	92 (94.8)	<0.01
Yes	17 (17.5)	5 (5.2)	
Exposure to arsenic			
No	85 (88.5)	90 (92.8)	0.31
Yes	11 (11.5)	7 (7.2)	
Source of milk intake			
Fresh milk	84 (86.7)	74 (76.3)	0.15
Tetra-pack	8 (8.2)	16 (16.5)	
Powdered milk	1 (1.0)	4 (4.1)	
Mixed source	4 (4.1)	3 (3.1)	
Water source			
Tap water	64 (66.0)	71 (73.3)	0.06
Hand pump	20 (20.6)	11 (11.3)	
Mineral/filter	8 (8.2)	13 (13.4)	
River	5 (5.2)	2 (2.0)	
Gender			
Male	67 (69.1)	67 (69.1)	1.00
Female	30 (30.9)	30 (30.9)	
Age group			
<16	46 (47.4)	46 (47.4)	1.00
16–29	40 (41.3)	40 (41.3)	
≥30	11 (11.3)	11 (11.3)	
Type of residence			
Urban	77 (79.4)	77 (79.4)	1.00
Rural	20 (20.6)	20 (20.6)	
Education level			
No education	19 (19.6)	19 (19.6)	1.00
Primary	40 (41.3)	40 (41.3)	
Secondary	24 (24.7)	24 (24.7)	
Higher	14 (14.4)	14 (14.4)	
Marital status			
Unmarried	72 (74.2)	72 (74.2)	1.00
Married	25 (25.8)	25 (25.8)	
Ethnicity			
Urdu speaking	41 (42.3)	27 (27.8)	0.04
Sindhi speaking	13 (13.4)	27 (27.8)	
Punjabi speaking	20 (20.5)	22 (22.7)	
Pashtun speaking	15 (15.5)	8 (8.2)	
Balochi speaking	6 (6.2)	9 (9.3)	
Others	2 (2.1)	4 (4.2)	

*p-value calculated by using the chi-square test

### Exposure to pesticides

Univariate logistic regression (model 0) showed that exposure to pesticides was significantly associated with AA cases (OR = 3.91, 95 % CI 1.38–11.07, *p*-value 0.01). In model 1, when we adjusted for the type of milk consumed and source of drinking water, the pesticide exposure was statistically significantly associated with AA (OR = 3.64, 95 % CI 1.03–12.81, *p*-value 0.04). In model 2, we adjusted for all covariates including exposure to arsenic and found that pesticide exposure remained a significant risk factor for developing AA (OR = 3.66, 95 % CI 1.04–12.88, *p*-value 0.04) (Table 4). The PAR was calculated as 12.72 %.

**Table 4:** Risk estimates for aplastic anemia (AA) with exposure to environmental factors (*n* = 194)

Characteristics	Model 0		Model 1		Model 2	
	OR (95 % CI)	*p*-value	OR (95 % CI)	*p*-value	OR (95 % CI)	*p*-value
Exposure to pesticides (organophosphates/DDT/insecticides/mosquito repellent)						
No	1		1		1	
Yes	3.91 (1.38–11.07)	0.01	3.64 (1.03–12.81)	0.04	3.66 (1.04–12.88)	0.04
Exposure to arsenic						
No	1		1		1	
Yes	1.64 (0.60–4.43)	0.76	0.95 (0.29–3.06)	0.94	0.88 (0.26–2.97)	0.84

Model 0 = univariate analysis

Model 1 = model 0 + water source + source of milk intake

Model 2 = model 0 + model 1

### Exposure to arsenic

Univariate logistic regression (model 0) showed that exposure to arsenic was not significantly associated with AA cases (OR = 1.64, 95 % CI 0.60–4.43, *p*-value 0.76). Similarly, after adjusting for other variables, i.e., ethnicity, type of milk consumed, source of drinking water and exposure to pesticides, no association was found between exposure to arsenic and AA cases (OR = 0.88, 95 % CI 0.26–2.97, *p*-value 0.84) (Table 4).

## Discussion

The findings of this study indicate that AA is associated with a lower socioeconomic profile and environmental exposure to several toxic substances among the Pakistani population. Individuals who were exposed to pesticides were significantly more likely to be diagnosed with AA. Our study results are suggestive of the fact that besides host genetics, several other hemotoxic factors may contribute to an environmental etiology of AA (Montané et al. 2008).

We found that literacy, which is the attainment of formal education, was significantly protective against aplastic anemia, and the odds of reporting AA decreased significantly with increasing levels of education. In other words, the illiterate remained at higher risk of acquiring aplastic anemia. Further, rural residents were also found more likely to report AA compared with their urban counterparts. This finding further adds to the international evidence that a lower socioeconomic profile is a risk factor for AA (Issaragrisil et al. 1995; Malhotra et al. 2015). As noted by S. Issaragrisil et al., a lower socioeconomic profile may very well be acting as a surrogate measure for several of the environmental exposures that may have an etiological role in the development of AA (Issaragrisil et al. 1995). The illiterate and rural residents of the country may be exposed to several toxic substances, pathogenic agents or medications that may play a role in the development of AA. For instance, evidence related to a higher association of hepatitis infection with AA continues to pour in (Rauff et al. 2011; Shah et al. 2011, 2012). Knowing that such infections are the diseases of poverty may further add to the importance of recognizing the poor as a high-risk subpopulation for AA (Awofeso 2001; Engels and Savioli 2006). Thus, this calls for a deeper understanding of the specific characteristics of illiterate and rural Pakistanis to provide evidence related to the risk factors for AA.

Further, discussing the identified environmental toxic exposures, pesticides were found to be strongly associated with AA disease occurrence among participants, which echoes results from other studies (Ahamed et al. 2006; Prihartono et al. 2011). Most of the individuals in Pakistan are exposed to pesticides in either the drinking water or vegetables, fruits and other edible items with various concentrations above the WHO/FAO permissible limits. Being an agricultural country, a 1169 % increase has been recorded with the use of different types of pesticides in the last 2 decades, and an almost similar rise in the burden of diabetes (Azizullah et al. 2011; Tariq et al. 2007). Studies report that occupational exposure to pesticides among farmers is strongly associated with all hematopoietic cancers (Merhi et al. 2007). Additionally, even the general population living in areas with extensive agricultural operations have high exposure to pesticides (Tahir and Anwar 2012). It is important to note here that our study results indicate that rural residents had double the risk of acquiring AA as compared to their urban counterparts. Although pesticide contamination of food and water and domestic use of pesticides were the main exposures assessed in our study, occupational exposures among the predominantly agrarian rural population in Pakistan cannot be ruled out. We therefore call for further evidence from the Pakistani context in this regard.

Arsenic exposure caused by soil and ground water contamination has remained a serious health concern for populations globally and has been related to various cancers and genetic and metabolic dysfunctions in humans (Shankar et al. 2014). The situation is a major public health concern in Pakistan as well, where contamination of drinking water in affected areas exceeds the WHO permissible limits (Bahadar et al. 2014; Rahman et al. 2009). Although we found this to be significantly associated with AA disease occurrence in the univariate analysis, after careful adjustment with important covariates, the significance of an association between arsenic exposure and AA eventually diminished. Nevertheless, arsenic substantially affects large population subgroups, and further evidence is warranted to explore its toxic effects on the etiology of blood disorders (Subhani et al. 2015). On subgroup analysis, the association of exposure to arsenic with AA was not statistically significant; this may perhaps have been due to the smaller number of exposed individuals in the subgroup analysis.

The findings of our study may provide useful information regarding environmental exposures to certain chemicals among patients with aplastic anemia. The study was conducted at the NIBD, which may be regarded as paramount in its expertise, and it caters to a large number of patients with blood disorders. Further, the sample selection of cases and controls was finely matched according to age and gender, also adding to the strengths of the study findings. Nevertheless, there are several limitations. First, exposure data were collected retrospectively, so there a chance of recall and information bias may remain. Second, despite the fact that there is a significant association of aplastic anemia with environmental exposures, the case-control nature of study limits the ability to establish a temporal association. However, despite the above-mentioned limitations, this study has provided useful information regarding the sociodemographic- along with lifestyle-related environmental exposure in acquired aplastic anemia. The information may be helpful in building evidence related to environmental risk factors for AA.

## Conclusion

This study observed a significant association of aplastic anemia with a lower socioeconomic profile and certain environmental exposures. The evidence may be helpful in understanding the pathophysiology of aplastic anemia in the context of environmental exposures.
